# Impact of Stiffness and Cell-Binding Motif Availability on the Cell-Specific Response to Collagen-Based Macromolecular Materials

**DOI:** 10.1021/acs.biomac.5c01434

**Published:** 2026-05-25

**Authors:** Natalia Davidenko, Daniel V. Bax, Emma Hunter, Samir W. Hamaia, Jean-Daniel Malcor, Richard W. Farndale, Sanjay Sinha, Serena M. Best, Ruth E. Cameron

**Affiliations:** 1 Department of Materials Science and Metallurgy, 2152 University of Cambridge, Cambridge CB3 0FS, United Kingdom; 2 Department of Biochemistry, 2152 University of Cambridge, Cambridge CB2 1QW, United Kingdom; 3 Department of Medicine, Cambridge Stem Cell Institute, 2152 University of Cambridge, Cambridge CB2 0AW, United Kingdom

## Abstract

Although matrix stiffness is an important determinant of cell behavior, experimentally isolating mechanical cues from the surface chemistry is challenging. Here, intact collagen (Col I) or a constant density of collagen-derived cell-adhesive triple-helical peptides (GFOGER or GLOGEN) was deposited on surfaces with physiologically relevant stiffnesses. Equivalent integrin ligation on each surface decoupled stiffness from collagen-receptor ligation. The cell response was highly cell type-specific. Human dermal fibroblast (HDF) adhesion was largely insensitive to matrix stiffness, while cytoskeletal organization was promoted on stiffer substrates, equivalently for GFOGER and GLOGEN. Human umbilical vein endothelial cells (HUVECs) and human dermal microvascular endothelial cells (HDMECs) adhered and formed PECAM-1-containing cell–cell junctions preferentially on lower-modulus substrates. For HUVECs, this was independent of the coating peptide; however, HDMECs possessed greater PECAM-1-containing cell junctions on GFOGER over GLOGEN. These results offer new insights into the effects of stiffness *vs* integrin ligation on the cellular response to materials.

## Graphical abstract

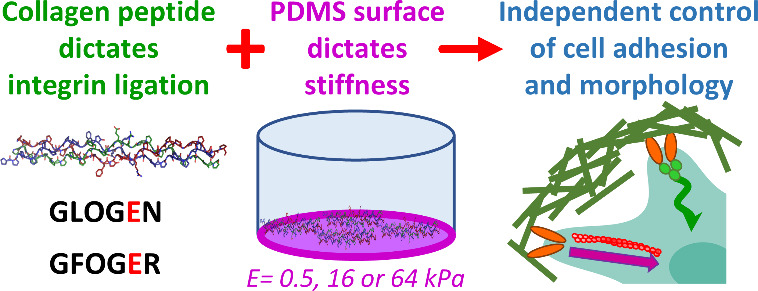

## 1 Introduction

The mechanical properties of the extracellular matrix (ECM) plays a significant role in determining the cell response,^1–4^ where cells reorganize their cytoskeleton, exerting tension onto cell-surface adhesive focal adhesions to activate signaling cascades.^5^ Cells perceive the mechanical environment via numerous pathways, including mechanical stretching of p130^CAS^, recruitment of vinculin to unfolded talin rod domains, FAK regulated signaling, and force-induced exposure of integrin-binding sites on extracellular matrix (ECM) proteins such as fibronectin.^5–8^ As such, small alterations in matrix stiffness can result in dramatic changes in cell behavior and phenotype^2,9,10^ including adhesion, differentiation, growth, motility, and survival.^2,3,11–14^ Stiffness, the resistance of a matrix to deformation, can be quantified through various metrics, one of which is Young’s elastic modulus (*E*), defined as the ratio of stress to strain. The stiffness of living tissues varies over many orders of magnitude: from several hundreds of Pascals (brain) to kilo-Pascals (10–20 kPa for muscles), mega-Pascals for tendon and cartilage, and giga-Pascals for bone, which is the same order of magnitude as tissue culture plastic.^10,15,16^ Disease is often associated with changes in tissue stiffness. For example the elastic modulus of liver increases from 300 to 600 Pa, when healthy, to 20 kPa for fibrotic or cirrhotic tissue.^15,17^

Determining the sensitivity of a cell to its surrounding matrix stiffness is experimentally challenging since modifications to the rigidity of a material often cause alterations in its structure and biochemical properties. Consequently, studies of matrix stiffness are often multivariable. A variety of model systems based on biopolymers, naturally occurring ECM proteins,^1,18–22^ or synthetic polymers^2,3,23,24^ have been investigated to isolate the effect of matrix mechanics on the cellular response. Among ECM-derived components, collagen, the most abundant natural polymer in the body, has been explored widely in these *in vitro* models.^1,21,25–27^ Collagen comprises a family of genetically distinct molecules with a unique triple-helical conformation.^28,29^ The most prevalent is fibrillar collagen type I, which provides structural support for many organs and tissues. Collagen contains multiple cell-ligation motifs that bind to cell surface integrins α1β1, α2β1, α10β1, and α11β1 ^30–32^ and nonintegrin receptors such as discoidin domain receptors (DDRs), glycoprotein VI (GPVI), human osteoclast-associated receptor (OSCAR), and secreted protein acidic and rich in cysteine (SPARC).^33,34^ Cell-collagen binding via integrins is sensitive to the triple-helical conformation of collagen. Hence, triple-helical peptides are used to study native cell interactions with collagen, identifying a consensus Gxx′GEx″ integrin binding sequence in collagen, encompassing the high-affinity GFOGER and GLOGEN motifs used here.^30,35^

Numerous approaches have been used to modify the mechanical properties of collagen-based materials. However, these tend to alter other material attributes simultaneously. As an example, chemical cross-linking, using carbodiimide hydrochloride (EDC), can effectively control the stiffness of collagen-based biomaterials over physiologically relevant magnitudes.^36–40^ Despite this, it has become apparent that this occurs at the expense of cell recognition, presumably through EDC-induced modification of cell-binding motifs.^38,40–42^ As such, it is not possible to assign a cellular effect to stiffness alone in this system. Alternatively, gel-like matrices were among the first systems demonstrating the significance of matrix mechanics on anchorage-dependent cell behavior.^20^ The stiffness of these gels can be modified, without altering its integrin recognition properties,^20,43,44^ by culturing them attached to a culture dish (thereby increasing tension/stiffness) or free floating. Although highlighting the importance of matrix stiffness, gel-like matrices are fragile and difficult to handle with issues of reproducibility, homogeneity, and cell imaging. These disadvantages can be circumvented by using thin films of fibrillar collagen I, where substrate stiffness is manipulated by altering the density of large collagen fibrils, or stiffening by dehydration.^1,25,26^ As the cell-binding sites on collagen are preserved, the effect of mechanics, against constant collagen ligation, can be obtained. However, thin films have restrictions in terms of their “tunability” of stiffness.

Instead, we have used well plates with specified rigidities (*CytoSoft*)^45^ consisting of a thin layer of biocompatible silicone with a range of physiologically relevant stiffnesses (from 0.2 to 64 kPa). The substrate modulus range of 0.5–64 kPa represents the native modulus of the majority of human tissues, from soft (0.5 kPa) brain to stiff (64 kPa) cartilage.^46^ These have been used previously to assess the effect of the mechanical microenvironment on different cell functions such as morphology and proliferation.^47–49^ However, in previous studies, no attempt was made to distinguish between the effects of changing stiffness and changing cell-ligating motifs. In this study, we address this important deficit by using cell-binding triple-helical peptides coated onto a range of *CytoSoft* well surfaces. GFOGER and GLOGEN were chosen as, although they bind to all four collagen-binding integrins, they do so with differing affinity, where GFOGER is a high affinity sequence for integrins α2β1/α11β1 ^50,51^ and GLOGEN is a high affinity sequence for α1β1/α10β1.^52,53^ This allowed us to change the availability of cell-ligating groups completely independently of matrix stiffness.

In this study, human dermal fibroblasts (HDF) and two types of endothelial cells (human umbilical vein endothelial cells, HUVEC, and human dermal microvascular endothelial cells, HDMEC), all expressing collagen-binding integrins, were selected to determine the effect of matrix stiffness versus cell-ligating sequences on the cellular response. These cell types were chosen as both are well-established cell models for the assessment of cell-biomaterial interactions.^2,54–58^ Cell adhesion, morphology, cytoskeletal assembly, and phenotypic marker localization were determined for cells cultured on differing collagen-derived cell-ligating sequences when coated onto surfaces possessing a range of stiffness. As collagen is of increasing interest for tissue engineering scaffolds,^31,32,59^ this assessment of the cellular response to stiffness *vs* bioactive motifs is vital to optimize the performance of macromolecular materials for a range of different applications.

## 2 Experimental Section

### 2.1 Materials

Insoluble microfibrillar collagen type I (Col I), derived from bovine skin, was purchased from Devro Medical Bathurst, NSW, Australia. 9-Fluorenylmethoxycarbonyl (Fmoc)-protected amino acids and *N*,*N*-dimethylformamide (DMF), used for peptide synthesis, were supplied by AGTC Bioproducts (Hessle, UK). Dulbecco modified Eagle's medium (DMEM), phosphate-buffered saline (PBS), fetal bovine serum (FBS), penicillin, and streptomycin were purchased from Invitrogen Life Sciences (UK). All other amino acids and reagents were purchased from Sigma-Aldrich (Gillingham, UK) at analytical grade.

GFOGER, GLOGEN, and GPP_10_ triple-helical peptides were synthesized using methods described in our previous work.^53,60,61^ Briefly, GPC­(GPP)_5_GFOGER­(GPP)_5_GPC (termed GFOGER), GPC­(GPP)_5_GLOGEN­(GPP)_5_GPC (termed GLOGEN), and GPC­(GPP)_10_GPC (termed GPP_10_) were assembled as C-terminal amides on Rink amide aminomethyl Tantagel resin (0.526 g, loading of 0.19 mmol g^–1^, RAPP Polymere, Germany) by an Fmoc/*tert*-butyl solid phase strategy. Cleavage from the resin was performed in a mixture of trifluoroacetic acid/triisopropylsilane/H_2_O 95/2.5/2.5 (v/v/v) with 250 mg of dithiothreitol for 2 h. The cleavage solution was concentrated and precipitated in cold diethyl ether. The precipitate was filtered, washed with cold diethyl ether, dissolved in H_2_O/acetonitrile 95/5 (v/v) (0.1% TFA), and freeze-dried. The crude product was purified by preparative reverse-phase high-performance liquid chromatography on a PerkinElmer LC200 system equipped with a 10 μm Eurospher II 100–10 C18 H (Knauer, Berlin, Germany). Purified compounds were characterized by matrix-assisted laser desorption ionization time-of-flight mass spectrometry.

### 2.2 Collagen and Peptide Coating of *CytoSoft* Well Plates

CytoSoft 6-well plates and CytoSoft Imaging 24-well plates with elastic moduli of 0.5, 16, and 64 kPa (CellSystems Biotechnologies, Vertrieb GmbH, Germany) were coated with 600 μL/well in 24 well plates or 3 mL/well in 6 well plates of 10 μg mL^–1^ Col I, GFOGER, GLOGEN, or control GPP_10_ peptides, diluted in 10 mM acetic acid, overnight at 4 °C. Volumes were calculated against the well surface area to maintain the coating density between multiwell formats.

### 2.3 Cells and Culture Conditions

#### Human Dermal Fibroblasts (HDFs)

HDFs were purchased from Sigma-Aldrich, UK, and maintained in a humidified incubator with 5% CO_2_ at 37 °C in DMEM, containing FBS (10% (v/v)) and streptomycin/penicillin (1% (v/v)). HDFs were subcultured at a ratio of 1:4 every 3–4 days. Cells were detached from the cell culture flasks with trypsin (0.05%)/EDTA (0.02%) (GE Healthcare), centrifuged at 180*g* for 6 min, and resuspended in serum-free DMEM. Serum-free media was used for cell analysis to exclude serum-derived cell-adhesive proteins, ensuring that adhesion was via the Col I or peptide coatings only.

#### Human Umbilical Vein Endothelial Cells (HUVECs)

Pooled HUVECs were purchased from PromoCell (Heidelberg, Germany) and cultured in Endothelial Cell Growth Medium 2 (EGM-2, PromoCell) at 37 °C with 5% CO_2_. HUVECs were subcultured at a ratio of 1:4 every 3–4 days. Cells were used at 70–90% confluence between passages 3 to 5. They were detached with tryplE for 5 min at room temperature, centrifuged at 280*g* for 4 min, and resuspended in EGM-2 for imaging analysis or serum-free DMEM for attachment assays.

#### Human Dermal Microvascular Endothelial Cells (HDMECs)

HDMEC from juvenile foreskin were purchased from PromoCell (Heidelberg, Germany) and cultured at 37 °C with 5% CO_2_ in Endothelial Basal Medium (EBM, PromoCell) supplemented with FBS (15% (v/v)) (Invitrogen), penicillin/streptomycin (100 U/100 μg mL^–1^) (Invitrogen), sodium heparin (10 μg mL^–1^), and bFGF (2.5 ng mL^–1^). HDMECs were subcultured at a ratio of 1:4 every 3–4 days. 70–90% confluent cells were used between passages 5 to 7. HDMECs were detached from the cell culture flasks with trypsin (0.05%)/EDTA (0.02%), centrifuged at 200*g* for 3 min, and resuspended in supplemented EBM for imaging analysis or serum-free DMEM for attachment assays.

### 2.4 Recombinant Integrin α1 and α2 I-Domains

Human I domains from T141 to E335 for integrin α1 and from S141 to E336 for integrin α2 were expressed as recombinant N-terminal Glutathione S-transferase (GST) fusion proteins as reported in our previous work^60^ and detailed elsewhere.^52,62^

### 2.5 Cell Adhesion on Col I/Peptide-Coated Surfaces of Different Stiffness

Cell adhesion in the presence of Mg^2+^ (integrin-mediated) or EDTA (nondivalent cation specific) was assessed calorimetrically using a lactate dehydrogenase (LDH) detection kit (Roche, Cat. No. 11 644 793001). LDH was deliberately released from bound cells by detergent lysis, thereby measuring an absorbance intensity proportional to the number of adherent cells.

Before cell addition, nonspecific adhesion to the coatings in 24-well plates was blocked with bovine serum albumin (BSA) (600 μL, 5% (w/v) in PBS) for 45–60 min followed by PBS washing (3 × 600 μL). Cell suspensions (400 μL) at the stated densities, containing either 5 mM MgCl_2_ or 5 mM EDTA, were added to wells and the cells allowed to attach for 60 min at room temperature. This duration was chosen to allow sufficient time for cells to settle onto and adhere to the Col I/triple-helical peptide-coated surface and to minimize time for cell-derived ECM synthesis that may contribute to cell adhesion. The wells were thoroughly washed with PBS (3 × 600 μL) to remove nonattached or loosely bound cells, and then lysis buffer (200 μL, 2% (v/v) Triton X-100 in distilled water) was added to each well for at least 120 min at room temperature. The cell lysate (50 μL) was pipetted into Immulon 2HB 96-well plates (Thermo Scientific), and an LDH detection substrate (50 μL), prepared according to the manufacturer’s instructions, was added to each well. Plates were incubated at room temperature until color had developed, typically from 10 to 30 min, and the absorbance read at 490 nm (A_490_) using a Fluostar Optima plate reader (BMG Labtech). Background adhesion was determined on BSA and GPP_10_ coated wells. Cell adhesion assays were performed in triplicate and values are reported as means ± standard deviations.

### 2.6 Collagen and Peptide Adsorption Detection

Col I and peptide-coated wells were blocked with BSA (3% (w/v)) in binding buffer (50 mM TRIS, 140 mM NaCl, pH 7.4 containing 1 mg mL^–1^ BSA) for 1 h at room temperature and then washed with binding buffer (3 × 600 μL). The mouse monoclonal anticollagen I primary antibody (clone COL-1, Sigma, C2456) and a goat polyclonal antimouse horseradish peroxidase (HPR) secondary antibody (DAKO) were used for Col I detection. For peptides, a single chain antibody to GPP repeats was selected as a primary antibody using the University of Cambridge phage library. Clone 13, used in this work, has a flag and His_6_ tag and showed high affinity to all triple-helical peptides.^63,64^ Anti-flag HRP was employed as a secondary antibody. Noncoated BSA-blocked wells were used as a negative control for Col I and peptides. The primary antibody (300 μL, 10 μg mL^–1^ in binding buffer) was added to wells and incubated for 1 h at room temperature. Wells were washed with binding buffer (3 × 600 μL) and subsequently incubated with of 1:10,000 diluted secondary antibody (300 μL) in binding buffer for 1 h at room temperature. The wells were washed with binding buffer (3 × 600 μL) and then a 3,3′,5,5′-tetramethylbenzidine (TMB) substrate (300 μL)­(Thermo Scientific) was added to each well and the reaction was stopped, after color had developed, by the addition of 2.5 M H_2_SO_4_ (300 μL) to each well. Solution from each well (100 μL) was pipetted into standard 96-well plates and the absorbance measured at 450 nm (A_450_) in a Fluostar Optima plate reader (BMG Labtech). This assay was performed in triplicate and values are reported as means ± standard deviations. Statistical significance was calculated using a Student *t* test.

### 2.7 α1- and α2-Integrin I-Domain Binding Assays

Plates were blocked with BSA (600 μL, 5% (w/v)) in binding buffer (50 mM TRIS, 140 mM NaCl, pH 7.4, containing 1 mg mL^–1^ BSA) for 1 h at room temperature. Following BSA blocking, the samples were washed with binding buffer (3 × 600 μL) and then incubated in the corresponding recombinant integrin α1- or α2-I domain (10 μg mL^–1^) in binding buffer containing either 5 mM MgCl_2_ or 5 mM EDTA. After 1 h of incubation at room temperature, the samples were washed with binding buffer (3 × 600 μL). The presence of the GST tagged recombinant I domain was detected by incubating with anti-GST-HRP conjugated antibody (400 μL, 1:10,000 diluted; Amershan GE Healthcare RPN1236) for 1 h at room temperature. The detection antibody was removed and the wells were washed with binding buffer (5 × 600 μL). The TMB substrate was added (400 μL), and the reaction was stopped by the addition of 2.5 M H_2_SO_4_ (400 μL). Solution from each well (100 μL) was pipetted into 96-well plates, and the absorbance was measured in a Fluostar Optima plate reader at 450 nm (A_450_). Values represent means of triplicates ± standard deviation. Statistical significance was calculated using a Student *t* test.

### 2.8 Immunofluorescent Analysis of Cell Structures

Immunofluorescent analysis was used to study cytoskeletal assembly and phenotypic marker localization on Col I and peptide (GFOGER and GLOGEN)-coated plates with different stiffness values (0.5, 16, and 64 kPa). For HDFs, cell shape, formation of vinculin-containing focal adhesions, and actin stress fibers were analyzed after 3 h of incubation at 37 °C with 5% CO_2_. For HUVECs and HDMECs, the cellular distribution of von Willibrand factor (vWF) and platelet endothelial cell adhesion molecule 1 (PECAM-1) was examined after 24 h. Coatings on tissue culture plastic (TCP) were included to compare to giga-Pascal rigidity.^15^ Nonspecific cell interaction with the underlying CytoSoft surface was blocked with BSA (3 mL/well, 2% (w/v) in PBS) for 45–60 min, and then wells were washed three times with 3 mL of PBS prior to cell seeding.

#### HDF Cytoskeletal Assembly and FA Formation

3 mL of cells at 0.3 × 10^5^ cells mL^–1^ (90,000 cells/well) were added to wells in a 6-well plate and incubated at 37 °C with 5% CO_2_ for 3 h. Cytoskeletal assembly and FA formation were limited to 3 h to minimize time for cell-derived ECM deposition to occur. Cells were fixed with formaldehyde (3.8% (w/v) in PBS) for 25 min, washed with PBS (3 × 3 mL), and then permeabilized in TritonX-100 (2 mL/well, 0.5% (w/v) in PBS) for 10 min. The samples were washed with PBS (3 × 3 mL), blocked with BSA (2 mL/well, 3% (w/v) in PBS), and then washed with PBS (3 × 3 mL) containing BSA (0.1% (w/v)). Subsequently, primary anti-vinculin antibody V9131 (1 mL/well, 1:400 dilution; Sigma-Aldrich) in PBS containing BSA (0.1% (w/v)) was added for 1 h. The primary antibody was aspirated, wells were washed with PBS (3 × 3 mL) and secondary antibody (1 mL/well, 1:500 dilution; ab175700 donkey anti-mouse AF567, Abcam, UK) containing Phalloidin-FITC (1:500; Thermo Fisher Scientific) was added for 1 h at room temperature. Samples were washed with PBS (3 × 3 mL), and the cell nuclei were stained with DAPI (1 mL/well, 1:20,000 in distilled water) for 5 min. The wells were washed with water (3 × 3 mL) and viewed using a Zeiss Observer Z1 fluorescent microscope.

#### Endothelial Cell Phenotype: PECAM-1, vWF, and Stress Fiber Staining

HUVECs or HDMECs (2 mL) were seeded on coated wells at two cell densities: subconfluent at 0.5 × 10^5^ cells mL^–1^ (100,000 cells/well) and confluent at 1 × 10^5^ cells mL^–1^ (200,000 cells/well) and incubated at 37 °C with 5% CO_2_ for 24 h. This 24 h incubation ensured sufficient time for PECAM accumulation at cell–cell junctions. Cells were fixed with formaldehyde (3.8% (w/v) in PBS) for 25 min, washed with PBS (3 × 3 mL), and then permeabilized in TritonX-100 (2 mL/well, 0.5% (w/v) in PBS) for 10 min. Samples were washed with PBS (3 × 3 mL) and blocked with BSA (2 mL/well, 3% (w/v) in PBS) for 1 h. After washing (3 × 3 mL, with wash buffer (WB) – PBS, 0.1% (v/v) Tween 20, 0.1% (w/v) BSA), rabbit anti-vWF (1 mL/well, 1:400 dilution in WB; abc6994, Abcam, UK), and mouse anti-PECAM-1 (1 mL/well, 1:500 dilution in WB; Santa Cruz Biotechnology, US), primary antibodies were added for 1 h. Wells were washed (3 × 3 mL/well, WB) then Alexa Fluor 568-donkey-anti-rabbit (1:500, ab17594, Abcam, UK), and Cy5-goat-anti-mouse (1:1,000, ab6563, Abcam, UK) secondary antibodies alongside Phalloidin-FITC (1:500) were combined in WB and added (1 mL/well) for 1 h. Wells were washed (3 × 3 mL) with WB and the cell nuclei were stained with DAPI (1 mL/well, 1:20,000 in distilled water) for 5 min. The wells were washed (3 × 3 mL) with water and viewed using a Zeiss Observer Z1 fluorescent microscope.

### 2.9 Quantification of Immunofluorescent Images

All image analyses were conducted using the CellProfiler software package. The pipelines for each analysis were as follows:

#### Cell Nuclei Quantification

Nuclei were identified from a grayscale DAPI-stained image using a global, Otsu, 2-class threshold. The total number of nucleus-associated objects was then counted.

#### Cell Aspect Ratio

Cell nuclei were identified from the DAPI-stained image as for cell nuclei quantification. The cell area was then found by propagation from the nuclei locations, on the corresponding grayscale actin-stained image, using a global, Otsu, 2-class threshold. The object dimensions were measured, and the cell aspect ratio was derived by dividing the maximum object length by the minimum object length.

#### FA Size Quantification

Cell nuclei were identified from DAPI-stained images as for cell nuclei quantification. Perinuclear staining was identified by propagation from nuclei locations on the corresponding grayscale vinculin-stained image using a global, Otsu, 2-class threshold. Separately, all vinculin was identified from the grayscale vinculin-stained image using a global, Otsu, 2-class threshold, using the same parameters as for perinuclear identification, except without propagation from the nuclei. Perinuclear vinculin was deducted from total vinculin to derive the FA-associated vinculin. The area of the vinculin-associated FA was then measured.

#### vWF Quantification

Cell nuclei were identified from the DAPI-stained image as for cell nuclei quantification. Cellular vWF staining was identified by propagation, from the cell nuclei locations, on the corresponding grayscale vWF image using a global, Otsu, 2-class threshold. Separately, total vWF was determined using a global, Otsu, 2-class threshold of the grayscale vWF image, without propagation from the nuclei location. Cellular vWF was subtracted from the total vWF to derive the extracellular vWF objects. The total area of the extracellular vWF-associated objects was measured.

#### Cell–Cell Boundary PECAM-1 Quantification

Nonspecific nuclear and perinuclear objects were identified from the grayscale DAPI and secondary-only stained images, respectively, using a global, Otsu, 2-class threshold. Separately, PECAM-1-associated objects were determined using a global, Otsu, 2-class threshold of the grayscale PECAM-1-stained image. Specific PECAM-1 staining was derived by subtracting the nonspecific nuclear and perinuclear-associated objects from the PECAM-1-associated objects. The total area of the specific PECAM-1-associated objects was then measured.

### 2.10 Statistical Analysis

Unless otherwise stated, all error bars indicate standard deviations. Statistical significance was determined with a Student *t* test with unequal variance where N/S indicates nonstatistically significant (*p* > 0.05), * indicates *p* ≤ 0.05, ** indicates *p* ≤ 0.01, *** indicates *p* ≤ 0.001, and **** indicates *p* ≤ 0.0001.

## 3 Results

### 3.1 Peptide/Col I Coating Density and Integrin-α1 and -α2-I Domain Affinity on Different Stiffness Substrates

Prior to the cell studies, the ability of the peptide-coated wells to isolate the effect of mechanics was tested. To do this, we verified whether changes in surface rigidities influenced (1) the coating density of collagen/peptides, thereby the amount of adhesive ligand available and (2) the exposure/conformation of adhesive sequences, which may impact the affinity of these adhesive sequences to cell-surface receptors. For this purpose, an Enzyme-linked Immunosorbent assay (ELISA) was carried out to detect the Col I/peptide density on 0.5, 16, and 64 kPa stiff surfaces (Figure 1). These show that similar amounts of Col I was detected on surfaces with different rigidities. A similar trend was observed for the triple-helical peptides. This indicates that changes in substrate stiffness did not affect the quantity of surface-bound biomolecules. Next, the receptor affinity was evaluated via the attachment of recombinant integrin-α1 and -α2-I domains to Col I/peptide coated surfaces, as these I-domains represent the integrin domains that binds to collagen.^30,65,66^

**Figure 1:**
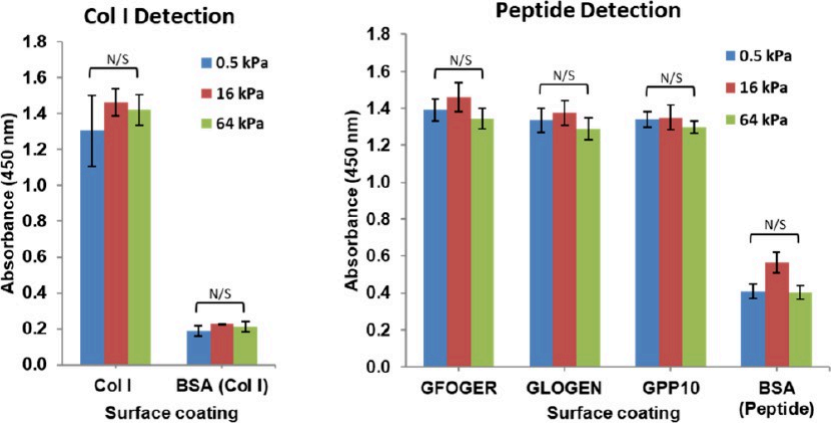
Col I and peptide detection on surfaces with different stiffness. Negative control values denoted by BSA (peptides) and BSA (Col I) represent background adsorptions on BSA-coated wells using the anti-peptide antibody and anti-Col I antibody, respectively. Note that different antibodies were used to detect Col I versus the triple-helical peptides, so direct comparison between the Col I and peptide absorbance values is not possible. Statistical analysis annotations: Bars with N/S show that no statistically significant differences were found among absorbance values for all peptides and Col I on different rigidity surfaces.

Integrin α1- and α2-I domain attachment was carried out in the presence of Mg^2+^ to measure integrin-mediated binding and EDTA for nonspecific adhesion. α1 (Figure 2A) and α2 (data not shown) adhesions in the presence of EDTA on all coatings were similar to the negative controls obtained on GPP_10_ coatings, demonstrating that I-domain adhesion is divalent-cation-dependent. Mg^2+^-dependent α1- and α2-I domain attachment to Col I, GFOGER, or GLOGEN was similar when coated onto surfaces with 0.5, 16, and 64 kPa stiffness (Figure 2B). This indicates that the intrinsic receptor-affinity of the adhesive sites in Col I and peptides was unaffected by underlying stiffness.

**Figure 2:**
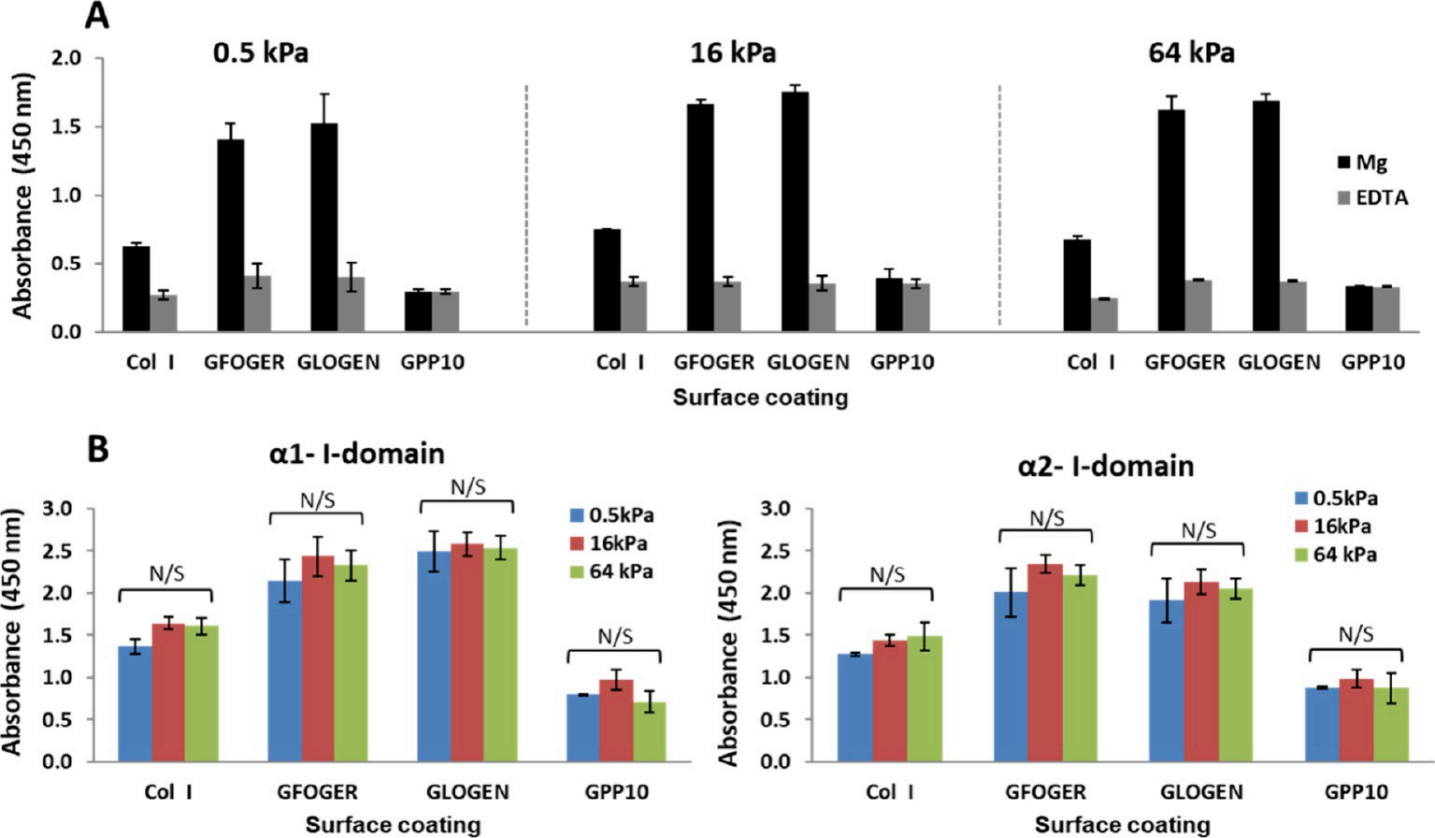
(A) α1-I domain adhesion to Col I/peptide-coated surfaces of differing stiffness in the presence of 5 mM Mg^2+^ (integrin-dependent) or 5 mM EDTA (nonspecific), showing that all binding is cation-dependent; (B) Mg^2+^-dependent α1- and α2-I domain adhesion to Col I and peptide-coated materials with different stiffnesses. Statistical analysis annotations: Bars with N/S show that no significant differences were found between Mg^2+^-dependent attachment levels of both I-domains to each peptide or Col I coating regardless of substrate rigidity.

### 3.2 Cell Adhesion to Col I and Peptide Coatings

No cell adhesion was noted in the presence of EDTA for all Col I and peptide coatings on all substrate stiffnesses, with absorbance values that were similar to the negative controls, BSA and GPP_10_ (data not shown). Therefore, all adhesion was deemed cation-dependent. Figure 3A–C shows magnesium-dependent HDF, HUVEC, and HDMEC cell adhesion to Col I and peptide coatings on different stiffness substrates, respectively. HDF adhesion was largely insensitive to changes in substrate rigidities or Col I/peptide coating (Figure 3A). However, adhesion of both HUVECs and HDMECs was influenced by substrate stiffness, but only with peptide and not Col I coatings (Figure 3B,C). For both peptide coatings, HUVEC and HDMEC adhesion was higher on substrates with lower stiffness, particularly 0.5 kPa surfaces compared to 16 and 64 kPa surfaces. By contrast, when Col I-coated, the attachment of both HUVECs and HDMECs was insensitive to changes in surface stiffness. On 16 and 64 kPa stiffness surfaces, both endothelial cell types adhered to a greater extent on Col I as compared with GFOGER and GLOGEN coatings.

**Figure 3:**
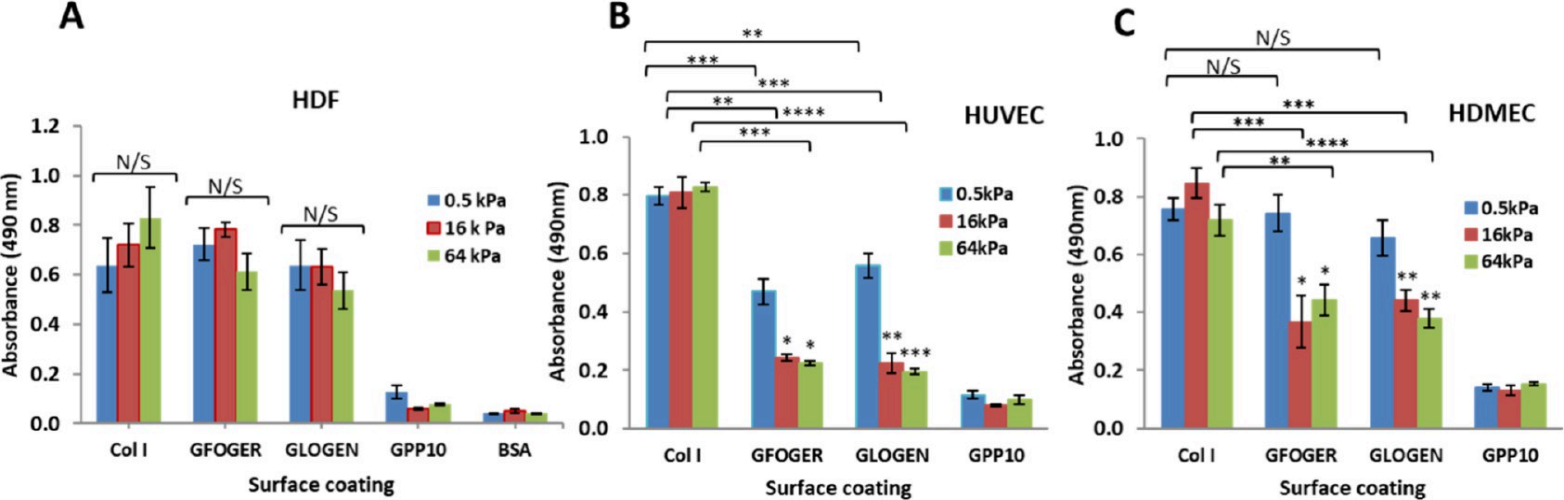
Mg^2+^-dependent adhesion to Col I and peptide-coated surfaces with different stiffnesses: (A) HDF; (B) HUVEC; (C) HDMEC. Statistical analysis annotations: (A) bar with N/S show that no statistically significant differences were found among attachment levels of HDF for any peptide or Col I coatings on all rigidities. (B, C) Bars with stars indicate the statistical differences between the data point on Col I and the corresponding peptide (GFOGER or GLOGEN) on surfaces of each rigidity. Annotations with stars alone show the statistical differences between the data point on 0.5 kPa surfaces and 16 or 64 kPa surfaces for each coating molecule.

### 3.3 Immunofluorescent Analysis of Cell Morphology

#### 3.3.1 HDF Cytoskeletal Structure

The aim was to examine the relative impact of substrate stiffness *vs* integrin engagement on fibroblast morphology, formation of vinculin-containing focal adhesion (FA), and actin stress fiber assembly (SF). Control polystyrene 6-well plates were included to compare with ubiquitously used giga-Pascal substrates. Cell nuclei quantification revealed similar cell numbers on different stiffness substrates regardless of the Col I/peptide coating (Figure 4Ai). HDF morphology, FA formation, and cytoskeletal assembly were qualitatively influenced by stiffness and coating molecules. Figure 4B shows that on Col I, HDFs are elongated, most probably due to contact guidance along the Col I fibers,^67^ whereas on peptides, the cells are more rounded, with a random spreading orientation. Quantification of the HDF cell aspect ratio (Figure 4Aii) agrees with the qualitative observations in Figure 4B, showing a significantly higher cell aspect ratio on Col I coatings compared to triple-helical peptides. Although the aspect ratio is independent of the substrate stiffness, other cytoskeletal features are qualitatively affected by the underlying stiffness. HDFs appear less stellate on 0.5 kPa surfaces in comparison with higher-modulus substrates. The images in Figure 4B show radial actin, with some thin SF and vinculin containing plaques, on peptide-coated 0.5kPa surfaces compared to thicker SF on Col I. On both Col I and peptide-coated 0.5 kPa surfaces, HDFs do not cluster vinculin with a few FA complexes at the ends of actin fibers (insert images in Figure 4B). This is particularly evident on peptide coatings. On more rigid, 16 and 64 kPa, surfaces, the cells form more actin stress fibers and more vinculin containing FA on the peptide-coated surfaces. This effect is markedly increased on coatings on standard tissue culture plates with stiffness in the gigapascal range (Figure 4B). Image analysis of FA size (Figure 4Aiii) agrees with this qualitative interpretation, showing that the vinculin-containing FA size on Col I coatings is not affected by the underlying substrate stiffness. By contrast, FA size on GFOGER and GLOGEN was influenced by the underlying substrate stiffness, where 0.5 kPa = 16 kPa < 64 kPa < TCP. Together, these observations indicate that fibroblasts noticeably change their cytoskeletal assembly on hard substrates where they form SF and FA structures with limited impact from the collagen-derived peptide coating.

**Figure 4:**
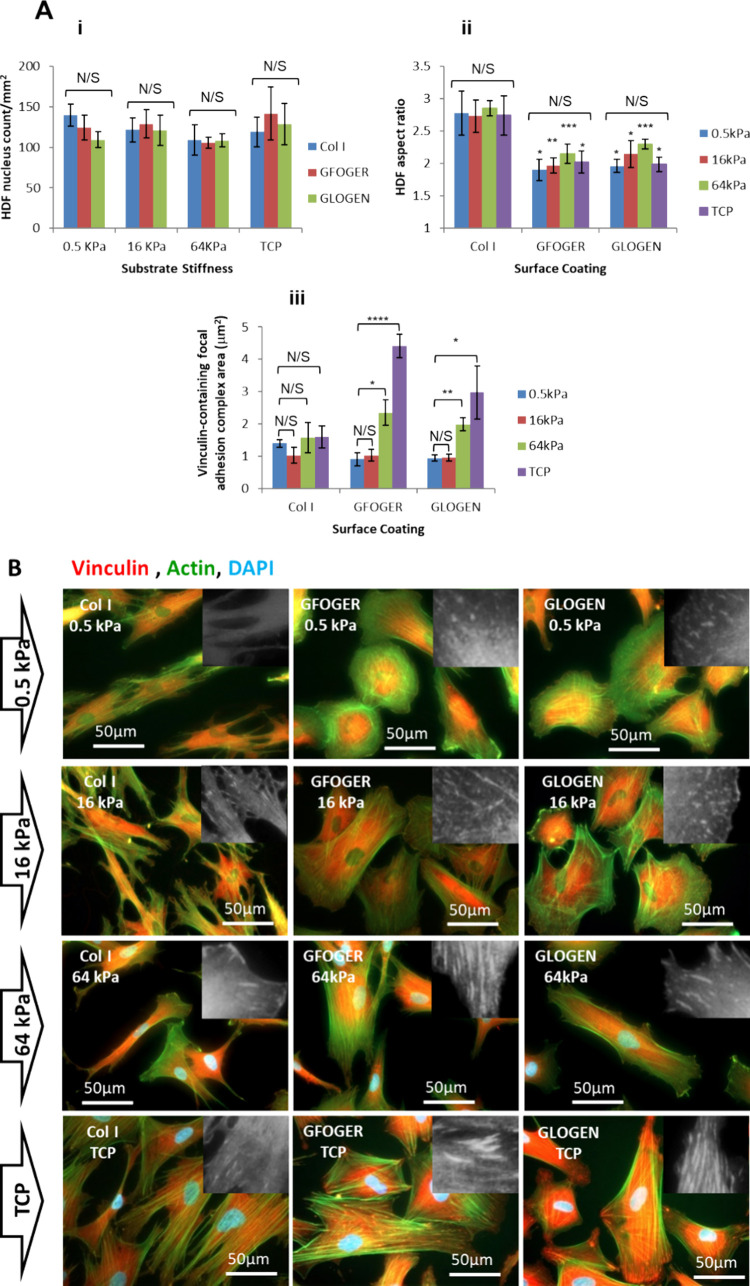
(A) Quantifications of (i) cell nuclei (ii) HDF aspect ratio and (iii) vinculin-containing FA area; (B) representative ×40 objective images of HDFs on Col I and peptide-coated surfaces with elastic moduli of 0.5, 16, and 64 kPa and standard tissue culture plates (TCP, with giga-Pascal rigidity). Inserts show vinculin-only staining of representative regions. Statistical analysis annotations: (i) bars with N/S show that there are no significant differences in the number of cells on all coatings and rigidities. (ii) Annotations with stars alone show the statistical differences between the data point on Col I and each peptide on surfaces of the same rigidity. Bars with N/S show that there are no statistically significant differences in HDF aspect ratio values for the same coating on all surface rigidities. (iii) Annotations show statistical significance between the stiffness indicated compared to 0.5 kPa for each surface coating.

#### 3.3.2 HUVEC and HDMEC vWF and PECAM-1 Distribution

The functionality of HUVECs and HDMECs was evaluated via staining of two specific markers of endothelial cell phenotype: vWF^68^ and PECAM-1.^69^ These studies were carried out at two cell densities: subconfluent (100,000 cells/well) and confluent (200,000 cells/well) to investigate the impact of cell–cell signaling and tension on the response to substrate stiffness. The images in Figure 5 show representative cell distributions of vWF (green) and PECAM-1 (red) in HUVECs and HDMECs seeded at subconfluence on Col I and peptide-coated surfaces of differing stiffnesses. These revealed similar vWF distributions irrespective of substrate stiffness and coating biomolecule.

**Figure 5:**
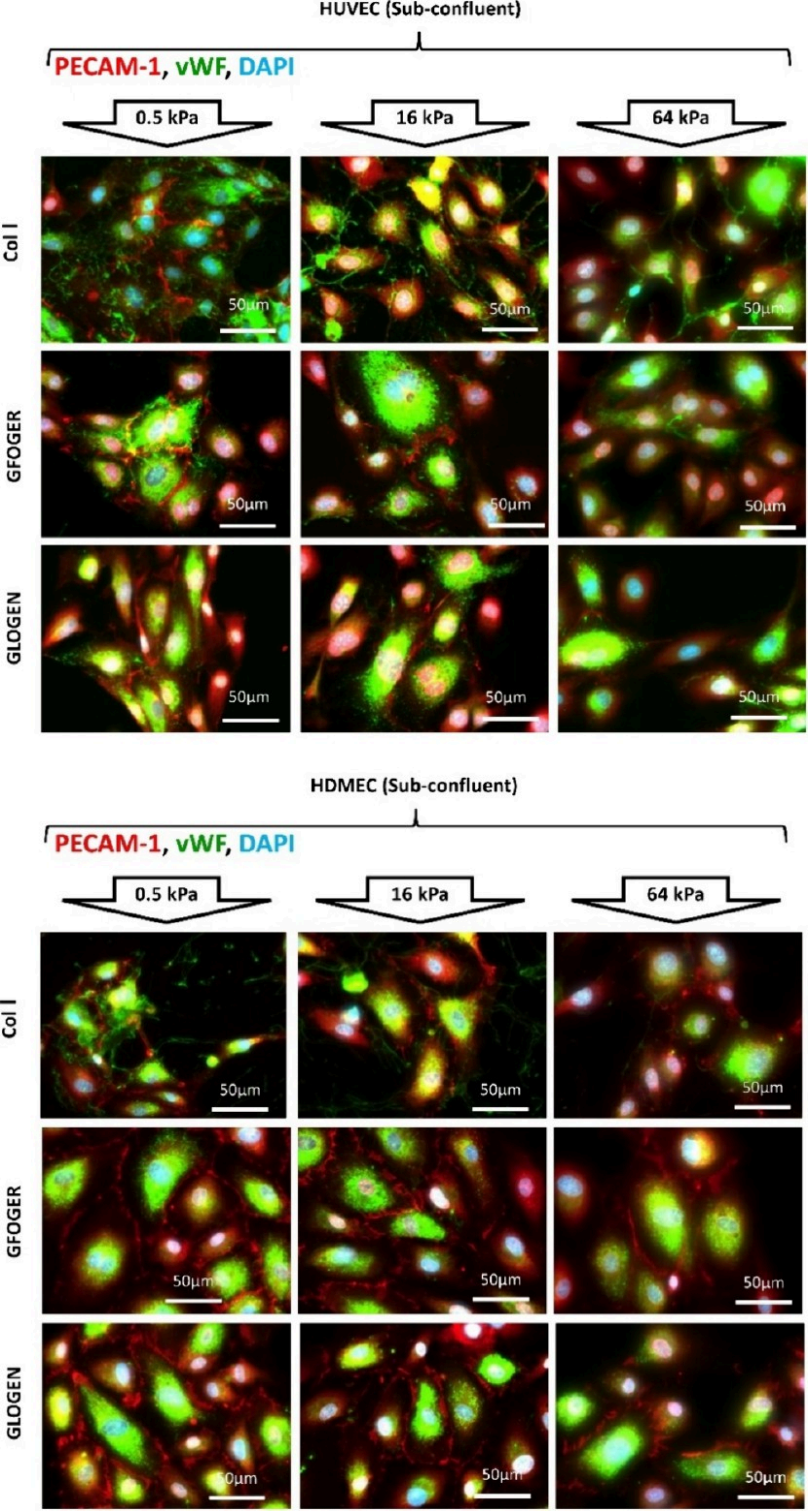
Representative ×40 objective images of HUVEC and HDMEC seeded at subconfluence on Col I and peptide coated surfaces with an elastic modulus of 0.5, 16, or 64 kPa. Cells were stained for endothelial cell markers: vWF (green) and PECAM-1 (red); and the nuclei are blue.

On Col I coatings, some of the vWF was observed on the extracellular collagen network as a green filamentous material. This was not present on the peptide-coated surfaces. This matrix-associated vWF was only observed in the proximity of cells (Figure 6A). On peptide-coated surfaces, both cell types seem qualitatively to produce equivalent vWF distributions on GFOGER and GLOGEN regardless of stiffness. All vWF was located in cellular vesicles, without any visible traces outside the cell. These qualitative observations were confirmed by quantification of the extracellular vWF (Figure 6B,C). This shows significantly elevated levels of extracellular vWF on collagen coated surfaces, compared to peptide-coated surfaces, for both cell types. For HUVECs, the amount of vWF on the collagen matrix was independent of the underlying stiffness, whereas for HDMECs, this decreased with increasing stiffness (0.5 kPa > 64 kPa). Cell nucleus quantification (Figure 6D,E) shows an approximately equivalent number of endothelial cells on all surfaces after 24 h in culture, except for HUVECs on GLOGEN where statistically higher cell numbers were found on lower modulus substrates (0.5 kPa > 16 kPa ≈ 64 kPa).

**Figure 6:**
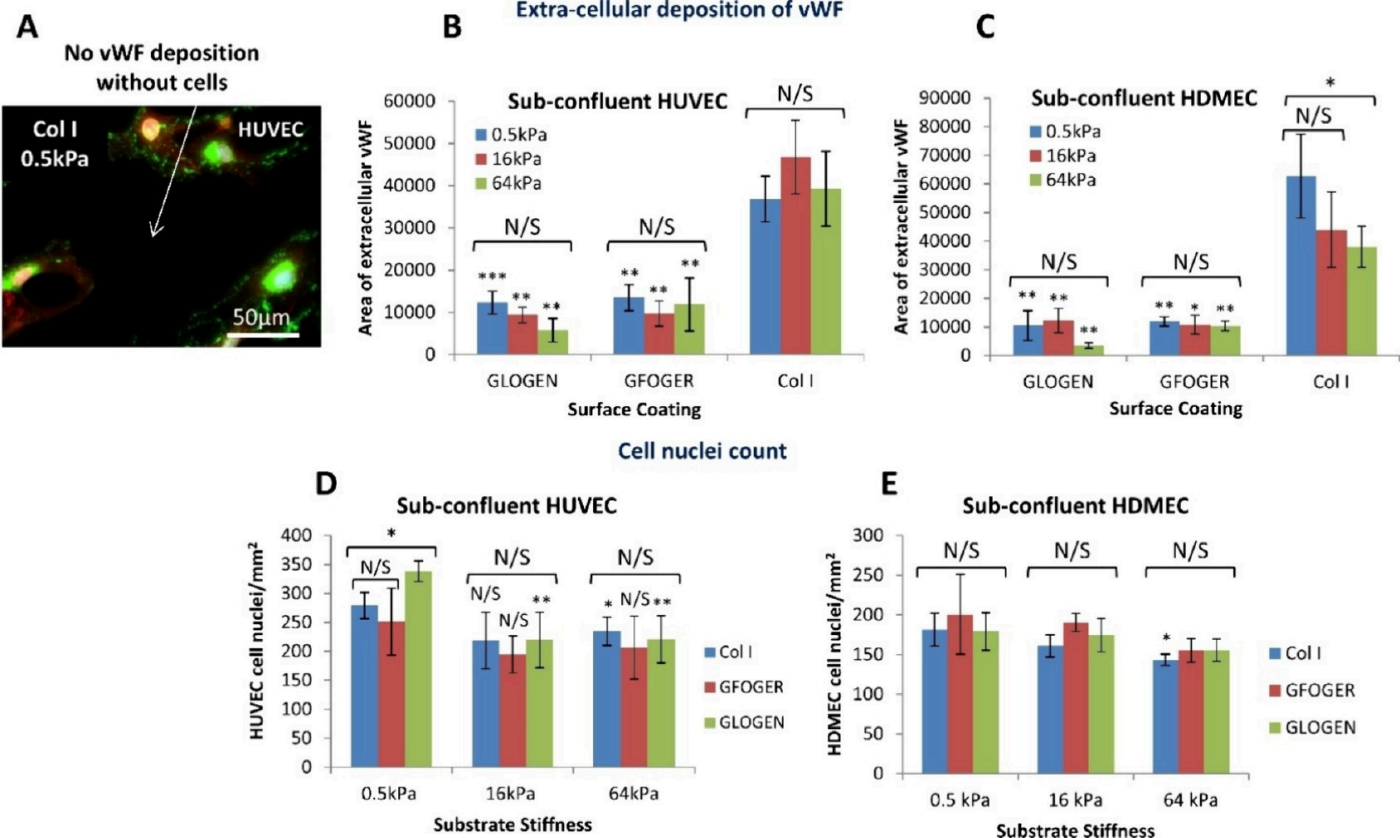
(A) Representative image of HUVECs on Col I coatings showing no extracellular vWF in the absence of cells; (B, C) quantification of extracellular vWF in HUVEC and HDMEC cultures, respectively; (D, E) cell nuclei quantification for HUVECs and HDMECs, respectively. Cells were seeded at 100,000 cells/well on collagen and peptide-coated surfaces with elastic modulus of 0.5, 16, and 64 kPa. Statistical analysis annotations: (B, C) annotations with stars alone show the statistical differences between the data point on Col I and each peptide on the surfaces of the same rigidity. (D, E) Annotations with stars or N/S alone show the statistical differences between the data point indicated and the equivalent coating on the 0.5 kPa surface.

Images in Figure 5 show differential localization of PECAM-1 to the cell–cell junctions in HUVECs and HDMECs depending on the substrate stiffness and Col I or peptide coating. These PECAM-1 distributions show qualitatively that HUVECs respond to substrate rigidity, localizing more PECAM-1 to the cell–cell connections on lower stiffness substrates than on 64 kPa substrates. HDMECs, however, seem to be relatively insensitive to changes in matrix stiffness, showing very similar profiles of peripheral PECAM-1 on surfaces with different rigidities.

Using imaging analysis, the level of PECAM-1 at the cell periphery was quantified for the HDMEC (Figure 7). A flowchart of the quantification process is presented in Supporting Information Figure 1. It should be noted that this analysis method was used to quantify the area of PECAM-1 located at the cell–cell boundary and not total cellular expression of PECAM-1, which would require quantitative analysis such as qPCR or FACS. This revealed that HDMECs were very responsive to the coating peptide but not to the rigidity of the underlying layers. The PECAM-1-containing cell–cell junction area followed the trend GFOGER > GLOGEN > Col I, independent of stiffness. This indicates a crucial role for peptide-integrin engagement in the localization of PECAM-1 to the cell–cell boundary.

**Figure 7:**
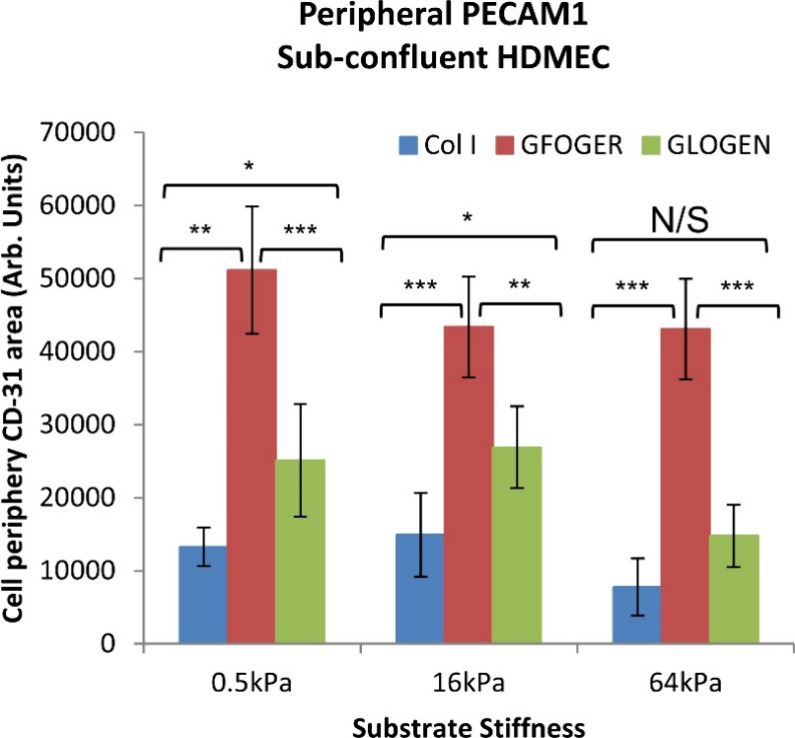
Peripheral PECAM-1 area quantification from HDMEC images taken on collagen and peptide coatings with elastic modulus of 0.5, 16, and 64 kPa. Cell seeding density was sub confluent. Statistical analysis annotations: Bars with stars show statistically significant differences between values of peripheral PECAM-1 area on Col I, GFOGER, and GLOGEN-coated surfaces of the same rigidity. The values of peripheral PECAM-1 area on the coatings of the same biomolecule coating are statistically equal for all rigidities (annotated statistics not shown).

A similar imaging study was carried out for HUVECs and HDMECs seeded at confluency (200,000 cells/well). Representative images (Figure 8) show that the majority of HUVECs on all biomolecule coatings and of HDMEC on peptides coatings were in close contact with adjacent cells. They were similarly extended and broadly covered these surfaces, regardless of stiffness. On collagen-based coatings, however, HDMECs were smaller and visually appeared to have a lower cell density in comparison to peptide-coated surfaces. Cell nucleus quantification (Figure 9A,B) confirms this qualitative observation, showing that the number of HDMECs decreased from ∼500 cells/mm^2^ on Col I coated 0.5 kPa surfaces to 280 and 220 cells mm^–2^ for Col I coated 16 and 64 kPa surfaces, respectively. On GFOGER and GLOGEN, however, the cell number was similar on all substrate rigidities (Figure 9B). For HUVECs, there were slightly more cells on the 0.5 kPa surface than on 16 and 64 kPa surfaces, especially with GLOGEN coatings (Figure 9A), similar to the trend that was found at sub confluence (Figure 6D).

**Figure 8:**
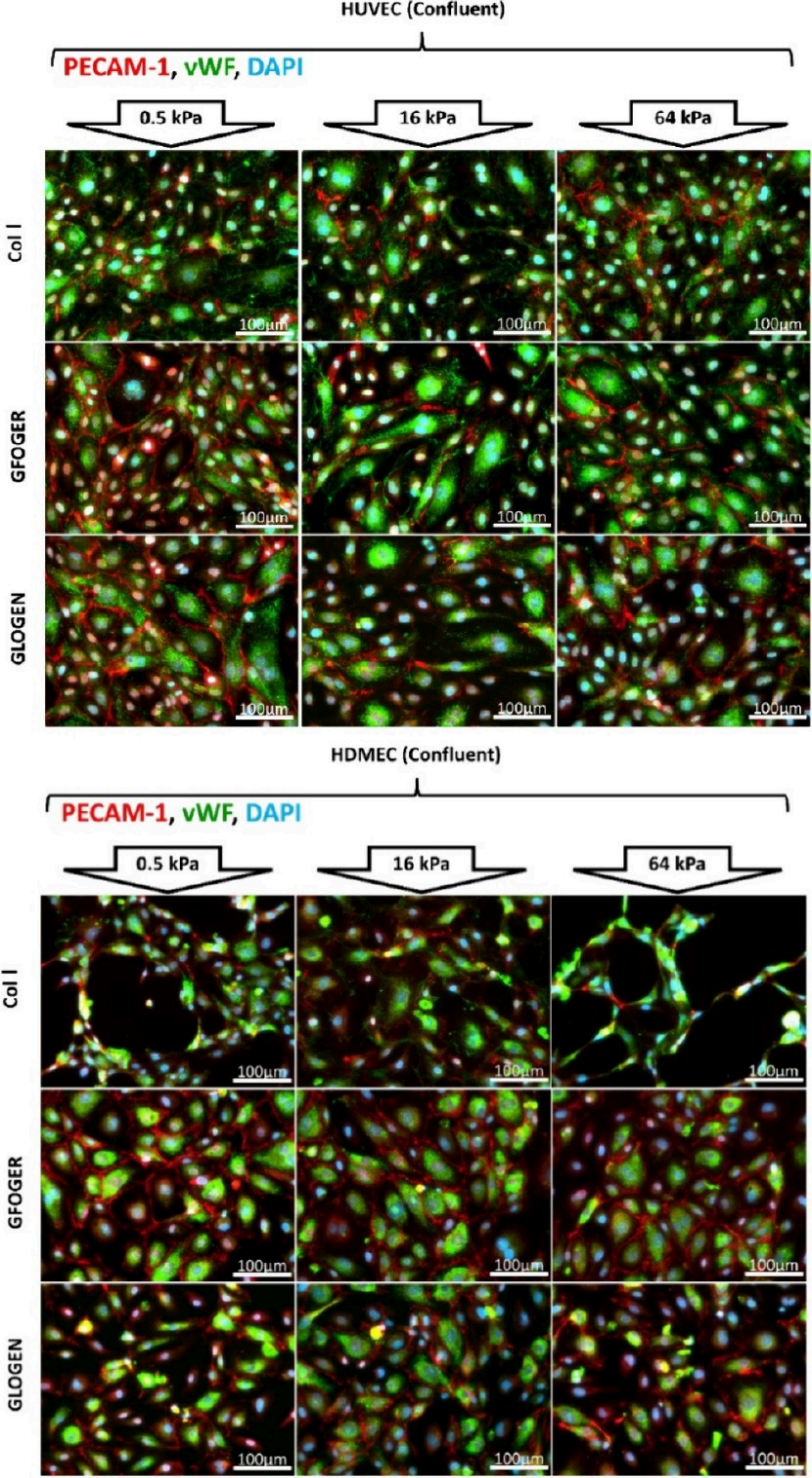
Representative images showing the distribution of PECAM-1 (red), vWF (green), and nuclei (blue) in HUVECs and HDMECs seeded at confluence (200,000 cells/well) on collagen and peptide coated surfaces with elastic moduli of 0.5, 16, and 64 kPa.

**Figure 9:**
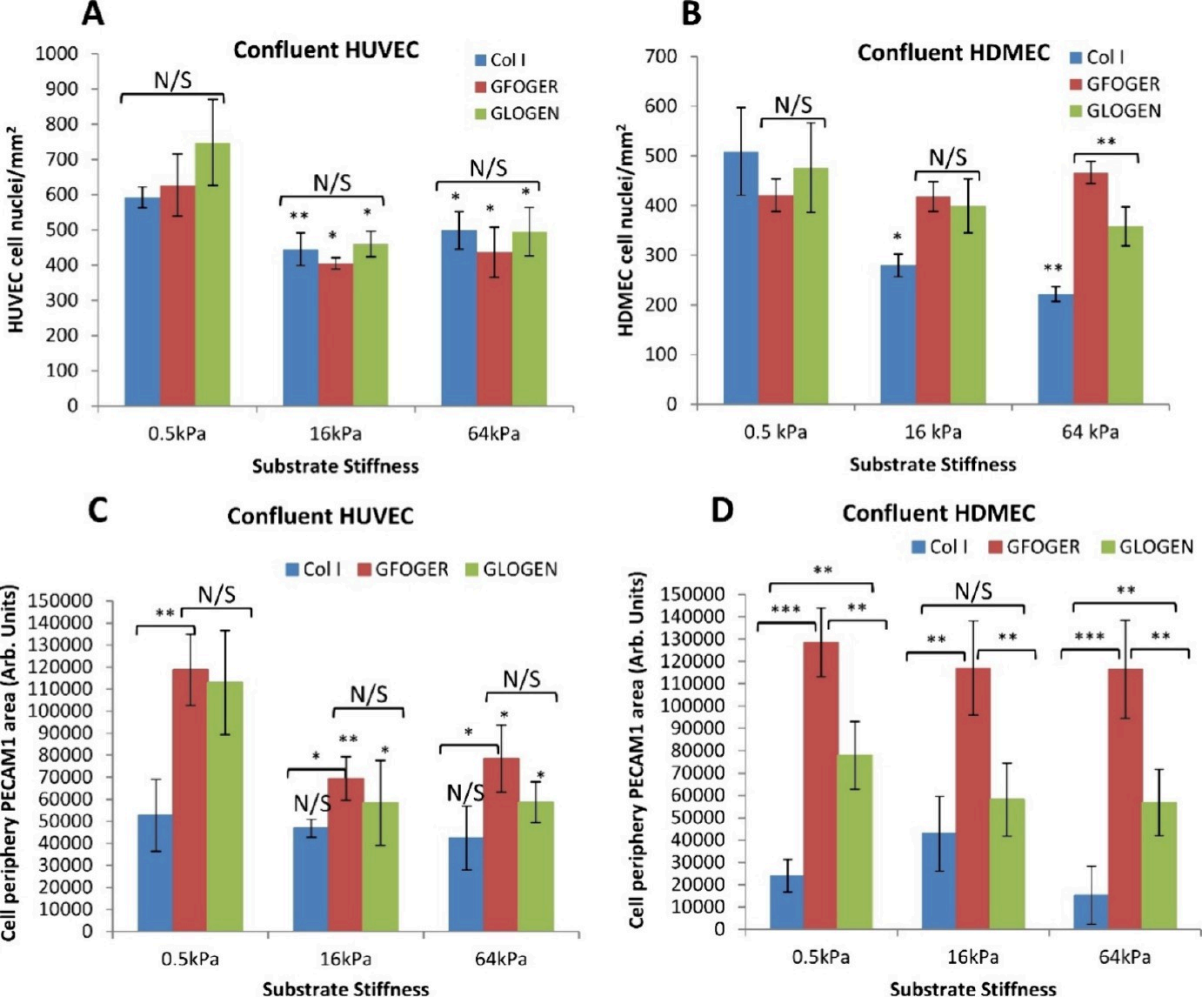
(A, B) cell nucleus quantification; (C, D) peripheral PECAM-1 area quantification for confluent HUVECs and HDMECs, on collagen and peptide coated surfaces with elastic moduli of 0.5, 16, and 64 kPa. Statistical analysis annotations: (A) annotations with stars alone show the statistical differences between the data points on 0.5 kPa samples and their stiffer (16 or 64 kPa) equivalents. Bars with N/S show that there are no significant differences in the number of cells on all coatings of the same rigidity. (B) Annotations with stars alone show the statistical differences between the data point on 0.5 kPa and stiffer (16 or 64 kPa) Col I coatings. There are no statistical differences between the data point on 0.5 kPa and stiffer (16 or 64 kPa) coatings for both peptides (not shown). (C) Annotations with stars or N/S alone show the statistical differences between the data point on the samples with lower stiffness (0.5 kPa) and their stiffer (16 or 64 kPa) equivalents. Bars with stars show the statistical differences between the data point on Col I and GFOGER surfaces with the same stiffness. Bars with N/S shows that there are no statistical differences between data points on GFOGER and GLOGEN of the same rigidity. (D) Bars with stars show the statistical differences between the data point on Col I and both peptides and between GFOGER and GLOGEN surfaces of the same stiffness. There are no statistical differences between data points for the same coatings of all rigidities (not shown).

For HUVECs, changes in surface rigidity did not visibly affect the distribution of vWF on all biomolecule coatings. The majority of the attached cells contained vWF vesicles, indicating that cell–cell contact at high cell density maintains vWF in HUVECs, independent of substrate rigidity. On peptide coatings, vWF was located in cytoplasmic vesicles in confluent cells, similar to that observed on subconfluent cells. On collagen coatings, some extracellular deposition of vWF can be observed on the collagen network; however, accurate quantification was impossible due to the small extracellular spaces between cells. Similar results can be observed for HDMECs, where little (if any) visible changes in vWF distribution could be found among surfaces of different rigidity.

Qualitatively, confluent HUVECs and HDMECs possess large areas of cell peripheral PECAM-1 on all substrate rigidities (Figure 8). The quantification presented in Figure 9C,D demonstrates that confluent HUVECs and HDMECs show different sensitivities to the underlying substrate stiffness and to the adhesive peptide. These figures suggest that, at confluence, HUVECs were responsive to stiffness, showing statistically higher PECAM-1 area at cell–cell boundaries on peptide-coated, low stiffness samples compared to their stiffer equivalents. This was independent of the peptide cell-binding motif sequence, i.e., GFOGER or GLOGEN. Col I surfaces promoted a lower cell-peripheral PECAM-1 area than on peptide-coated surfaces, which was unaffected by the substrate stiffness. The confluent HDMECs showed a similar trend of peripheral PECAM-1 location when compared to subconfluent HDMECs (Figure 7). Peripheral PECAM-1 at cell–cell boundaries in HDMECs was not sensitive to the substrate rigidity but instead was dependent on the coating biomolecule (Figure 9D). Significantly larger areas of PECAM-1-containing cell–cell connections were observed on peptide-coated surfaces than on Col I, with GFOGER sequences promoting maximal peripheral PECAM-1 area in HDMECs. This indicates that the signaling induced by GFOGER is essential to form PECAM-1-containing cell–cell junctions in HDMECs.

HUVEC’s assembled actin stress fibers on both GFOGER and GLOGEN coatings when seeded at confluence (Figure 10). By comparison, when adhering to Col I, fewer stress fibers were observed. Qualitatively, there was little impact from substrate stiffness for the peptide coatings. Therefore, stress fiber assembly of HUVECs, at high density, was less sensitive to substrate mechanics compared to HDFs (Figure 4).

**Figure 10:**
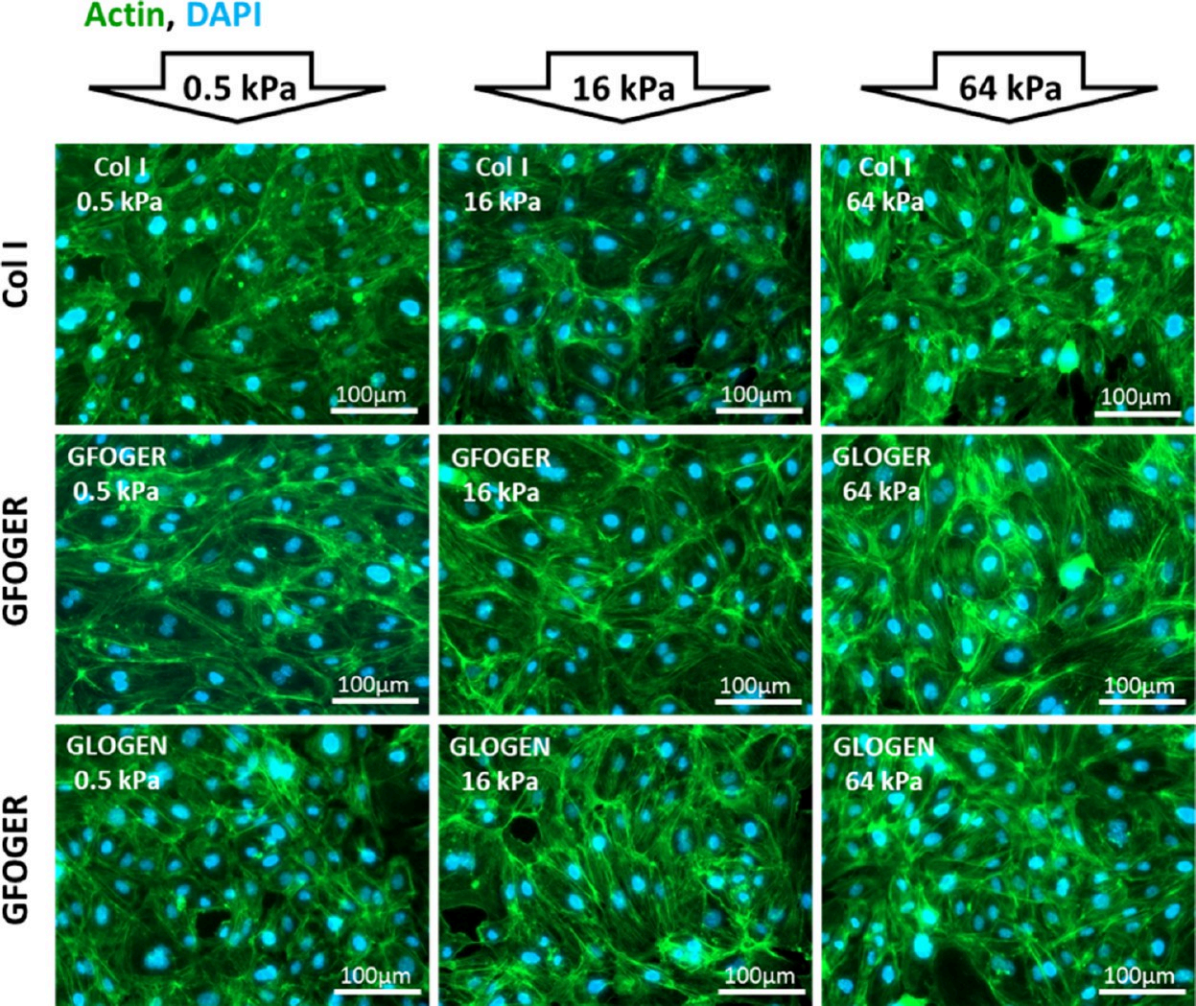
Representative images showing the distribution of actin (green) and nuclei (blue) in HUVECs seeded at confluence (200,000 cells/well) on collagen and peptide coated surfaces with elastic moduli of 0.5, 16, and 64 kPa.

## 4 Discussion

The mechanical and cell-receptor ligating properties of the matrix are critical in determining the cell response. However, isolating and quantifying the effect of mechanical cues versus receptor ligation on the cellular response is experimentally challenging. This is because changes in substrate rigidity are often associated with changes in the structure or biochemical properties of the matrix with consequent multivariable impact on cell behavior. In this work, we ensured consistent cell-ligating activity through fibrillar Col I and synthetic triple-helical peptides containing the cell-adhesive motifs GFOGER or GLOGEN. These were immobilized onto surfaces of known, physiologically significant rigidities, from several hundreds of Pascals (brain) to kilo-Pascals (10–20 kPa for muscles), and giga-Pascals for bone. In doing so, we could measure the impact of receptor binding versus material stiffness independently. GFOGER and GLOGEN were compared to intact collagen molecules as they are high affinity motifs for all four collagen-binding integrins; α1β1, α2β1, α10β1, and α11β1. As these peptides represent a small portion of the intact collagen molecule, they can bestow collagen-cell binding activity to the substrates while not influencing the bulk material mechanical properties. Flanking, five GPP sequences and one GPC triplet, on both sides of the cell-recognition motif ensured the triple-helical configuration and material surface association of these peptides.^53,61^

As our premise is to isolate cell ligating from surface mechanics, it was important that other cell-influencing factors were controlled. These factors include the absorption level of protein/peptides, which could directly affect the density of cell-recognition ligands available for interactions with cell receptors, and the exposure/conformation of these ligands, which may alter ligand affinity. Antibody detection showed statistically similar levels of collagen bound to surfaces of all stiffnesses. Similarly, the quantity of bound peptides was not sensitive to the surface stiffness, so the underlying rigidity does not appear to significantly affect the surface biomolecule coverage. It should be noted that, as different antibodies were used to detect Col I and the triple helical peptides, it is not possible to compare the amount of Col I and peptides bound directly, only that the surface rigidity does not affect the binding of each molecule individually. Indeed, due to the smaller mass, it would be predicted that a higher surface coverage of the triple helical peptide is possible. CytoSoft plates were chosen as the PDMS layer is chemically modified by the manufacturer to possess surface anhydride groups. These can covalently link to the amino terminus or the secondary amines on the prolines in the GPS­(GPP)_5_ sequences that flank the integrin-binding GFOGER or GLOGEN motif. There are 66 prolines in total in these flanking sequences, which can covalently link to the CytoSoft surface without chemically modifying the integrin-binding motif. This covalent linkage prevents surface dissociation of the peptides.

The properties of the CytoSoft surfaces were not verified here as they are provided precoated as thin films on multiwell plate surfaces, rendering such analysis unfeasible. PDMS surfaces are typically hydrophobic with water contact angles of approximately 100°. However, recombinant integrin I-domains and cells do not directly contact the PDMS surface in our assays as all surfaces are Col I or triple-helical peptide coated and any uncoated surface is passivated with BSA. Full BSA passivation is evident from the lack of cell adhesion to the control BSA-coated wells (i.e., in the absence of Col I or triple-helical peptide). Typically, BSA passivated surfaces have a water contact angle of 60–70°, which is likely the surface hydrophobicity experienced by the cells.

Surface association can induce conformational changes that can, in turn, influence affinity toward cell receptors. As this is surface-specific, the receptor affinity of the bound Col I and peptides was tested with isolated cell-adhesive receptors. Recombinant α1- and α2-I domains were chosen as the principal collagen interactive domains within integrins α1β1 and α2β1, respectively, via coordination with a Mg^2+^ ion.^30,65^ We found that I-domain affinity was not affected by the rigidity of the underlying material, indicating that the conformation of the triple-helical ligands had not been altered, certainly not to an extent that it impacts on integrin binding. Together, these results indicate that Col I/peptide-coated surfaces are suitable for studying force-induced versus biochemical-induced cell responses independently.

HDF and two types of endothelial cells, HUVECs and HDMECs, were used since they are sensitive to force-induced factors in a cell-type-specific manner.^2,10,15^ Fibroblasts, in particular, have been used to study the effect of substrate mechanics on cell function,^2,70,71^ allowing us to compare our results with those reported in the literature. Cell adhesion is dependent on multiple factors including (1) the density of cell-recognition ligands; (2) the affinity of the cell receptors to these sequences; and (3) the density of the adhesive receptors on the cell surface. By confirming identical density and affinity of our peptides for all surface stiffnesses, we have shown that the first two parameters were unaltered by the surface stiffness and, as such, did not contribute to changes in the cell adhesive response to matrix mechanics. The cell surface receptor density was maintained by using the same cell preparation for all conditions within the adhesion assay. Therefore, the sole variable was the substrate stiffness of the cellular response. Equivalent recombinant integrin I-domain binding to peptides on all substrate moduli discounts surface-induced deactivation of the triple-helical peptide activity. This integrin I-domain binding assay has an equivalent duration to the cell adhesion and HDF cytoskeletal assembly assays. Therefore, we presume that there is a limited surface-induced loss of peptide activity during cell analysis. Use of a stringent washing regime post cell adhesion ensured that only high-affinity interactions were measured. Integrin dependency during cell adhesion was monitored by ETDA inclusion to chelate the divalent cations from, and hence deactivate, cell-surface integrins. EDTA ablated cell adhesion to Col I and triple-helical peptides, presumably by inhibiting cell surface integrin binding to the integrin-binding motifs present in these molecules. All attachment assays and HDF cytoskeletal assembly assays were performed in the absence of serum. For PECAM and vWF immunofluorescence assays, a low serum (2% (v/v)) medium was chosen to preserve the endothelial cell phenotype. These conditions were used to avoid influence from adhesive (e.g., vitronectin or fibronectin) or proteolytic serum proteins.

Cellular adhesion to collagen-coated substrates was highly cell-type-dependent. HDF adhesion was largely insensitive to matrix stiffness whereas both types of endothelial cells clearly attached more to lower stiffness surfaces. This cell response to stiffness was more apparent on peptide coatings than insoluble Col I, being particularly evident for stiffer, 16 and 64 kPa, surfaces where more adhesion of both HUVECs and HDMECs was noted on Col I over peptides. This reduced sensitivity to the substrate rigidity when coated with insoluble Col I could be due to the cushioning effect of collagen fibrils, where the cells are responding to the mechanical properties of the collagen fibrils and not the underlying material stiffness. This may be because cells interrogate their environment on a very local (submicron) level.^1,2,15^ The triple helical peptides are approximately 1.5 nm thick. As just-saturating concentrations of peptide were used to coat the surface, we presume a 1.5 nm thick layer of peptide is present. By contrast, insoluble collagen has a very wide range of sizes, typically 1–20 μm. Once blended and surface-coated, insoluble collagen fibrils extend approximately 600 nm above the surface.^26^ Therefore, the insoluble collagen layer is potentially 200-fold thicker than the peptide coating, so cells may only experience the ColI fibril stiffness and not the underlying surface modulus. The thin peptide layer, by contrast, would more directly confer the surface stiffness to the cells, so they sensed the underlying material modus. This highlights the difficulty of decoupling mechanical versus biochemical properties using intact collagen, validating our peptide coating approach. The influence of rigidity on the ligation properties of HUVECs and HDMECs to peptide-coated surfaces implies that the formation of initial adhesion complexes between cell integrin receptors and collagen cell-binding sequences is dependent on mechanical forces exerted at cell attachment points. However, more studies are required to elucidate the roles of specific integrins and cell signaling pathways in transducing or mitigating responses to mechanical cues at the early stages of integrin–matrix interaction.

Cell morphological studies were used to further investigate the influence of the surrounding mechanical environment on the response of different cell types. For HDFs, we investigated the cellular aspect ratio, cytoskeletal organization, and formation of vinculin-containing FA structures. This is because FA plays a pivotal role in the mechanosensing of matrix environment,^15,72,73^ typically being formed in response to mechanical forces that the cell exerts on the matrix within approximately 1 h of surface contact.^1^ We conducted immunofluorescent analysis after 3 h of cell incubation to ensure sufficient time for the cells to respond to the surface stiffness. These showed that cell morphology, cytoskeletal assembly, and FA formation were influenced significantly by matrix rigidity in conjunction with biomolecule coating. On all Col I coatings, cells were more elongated and extended along collagen fibers, whereas on peptide-coated surfaces, cells were more rounded and randomly orientated. This is presumably due to contact guidance cues from the insoluble collagen fibrils, orienting cells along their length. Substrate stiffness was an important determinant of the HDF cell cytoskeletal organization, with increasing substrate stiffness inducing a stellate morphology and the development of actin SF and vinculin-containing FA complexes. Our results are consistent with numerous reports,^1,10,15^ showing that fibroblast morphology is dependent on substrate stiffness where the formation of SF and FA structures is promoted on a stiffer mechanical environment,^1,2,15,72,73^ although, unlike this study, these previous studies were not in combination with highly defined collagen receptor ligation. This role of mechanical tension in the development of SF and FA assemblies on collagen-based materials is consistent with others,^3,22,24^ showing that fibroblasts cultured in collagen floating gels rarely produced SF, but if these gels were anchored to the well surface, resulting in isometric tension, the cells formed SF. However, in these collagen gel-based studies, the mechanical properties of the material are less defined than presented here. As such, we have shown that stiffness in the range of 0.5 to 64 kPa influences cell morphology via collagen ligation. HDFs possess numerous collagen-binding receptors including integrins α2β1 and α1β1.^74^ This is consistent with our finding of a stiffness-associated increase in FA size on both GFOGER and GLOGEN peptides, presumably via ligation and mechanotransduction through these integrins. By comparison, SF formation in confluent HUVEC monolayers was relatively insensitive to substrate stiffness. This may be due to the continuous endothelial cell layer where cell–cell communication and tension may dominate versus the noncontinuous nature of the HDF monolayer where the cells are influenced predominantly by the substrate.

The impact of mechanical cues on endothelial cell functionality was assessed via immunofluorescent staining of two markers: vWF to identify endothelial cells and PECAM-1 (CD-31) as an indicator of the formation of cell–cell connections. Subconfluent and confluent seeding densities were employed to determine if signaling via direct cell–cell contact would change the receptiveness to mechanical forces. After 24 h, the vWF distribution in both HDMEC and HUVEC cells was similar. On 0.5 kPa matrices, almost all cells possessed vWF-containing vesicles, irrespective of the collagen-derived cell-binding motif. However, on stiffer, 16 and 64 kPa samples, especially in the case of Col I coatings, some cells did not possess vWF-containing vesicles, particularly when seeded at subconfluency. This may indicate that the formation of vWF-containing vesicles on Col I was subtly increased when coated with lower stiffness substrates. It was also apparent that the biomolecule coating plays an important role in the cellular location of vWF, where vWF is located both within the cell and on the extracellular fibrillar Col I coating. On peptide coatings, all vWF was confined to cytoplasmic vesicles as is characteristic for endothelial cells.^57,58^ The presence of extracellular vWF on Col I, but not peptide-coated surfaces, is consistent with the presence of the specific high-affinity composite site, GARGQAGVMGFO/GARGEOGNIGFO, that binds vWF, found in the alpha1/alpha2 chains of intact secreted collagen I. These are absent from the GFOGER/GLOGEN triple-helical peptides.

Cell number quantification, estimated by nucleus counts after 24 h, showed that at subconfluence, the overall cell number was minimally affected by the substrate mechanical properties. Confluent HDMEC monolayer cell numbers were only responsive to matrix rigidity on Col I coatings with decreasing cell numbers on stiffer matrices. Generally, cell counts after 24 h were neither altered by stiffness nor coating biomolecules. These tendencies are in apparent disagreement with the results of attachment tests after 60 min, where adhesion of both HDMEC and HUVEC was clearly higher on lower stiffness substrates. This may be attributed to short-term adhesion assays measuring solely strong, integrin-dependent cell engagement that resisted a stringent washing regime. By contrast, image analysis after 24 h did not include a washing procedure, and so all cells were quantified, regardless of initial affinity. This may imply that initial cell anchorage involving integrin–matrix engagement is sensitive to mechanical cues, whereas longer-term cells can populate the material, regardless of the mechanical properties. Alternatively, it is possible that cell-derived ECM deposition/remodeling renders the cells more adhesive and less sensitive to stiffness after 24 h in culture. Alternatively, cells were cultured for 24 h in serum-containing medium, whereas short-term adhesion assays were serum-free. Therefore, serum-derived adhesive proteins, such as vitronectin or fibronectin, may reduce the cell sensitivity to the underlying matrix stiffness for 24 h cultured cells, thereby explaining the difference in cell adhesion after short-term *vs* 24 h culture.

At subconfluence, HUVECs possessed an elevated PECAM-1-containing cell–cell junction area on lower stiffness substrates. It is unlikely that this effect was due to differing cell density on the different stiffness substrates as HUVECs formed similar sized cell clusters on all surfaces, but instead it appears due to HUVECs being receptive to the underlying material mechanical properties. PECAM-1 accumulation at cell–cell junctions is likely through diffusion trapping, where the relatively free movement of PECAM across the cell membrane is prevented through PECAM binding with its ligand on an adjacent cell.^75^ As such, PECAM-1 accumulation is a useful probe for tight cell–cell junction formation. Our observation, that substrate stiffness influences PECAM-1 accumulation at cell–cell boundaries, and by inference, the formation of tight-cell–cell junctions could be due to mechanotransduction through integrin α2β1, which plays a major role in the HUVEC cell response to collagen^76^ and is expressed at high levels in these cells. Additionally, HUVECs express integrin α10β1, albeit at lower levels than α2β1, so integrin α10β1 could contribute to the HUVEC response. In confluent HUVECs, this effect was not apparent with PECAM-1-containing cell–cell junctions equally visible in cells on surfaces of all rigidities. Although difficult to ascertain from the qualitative images, semiquantitative analysis of cell-peripheral PECAM-1 showed that confluent HUVECs were indeed responsive to matrix mechanics, especially when adhering to peptide-coated surfaces, showing increased cell-peripheral PECAM-1 on 0.5 kPa matrices. On Col I-coated surfaces, there was less cell-peripheral PECAM-1, which was unaffected by substrate stiffness, presumably due to the cells responding to the inherent stiffness of the fibrillar collagen matrix as opposed to the underlying substrate. Overall, HUVEC responsiveness to matrix stiffness was significantly diminished at confluency, indicating that cell–cell proximity is attenuating mechanosensing. Our supposition agrees with reports showing that cell sensitivity to mechanical forces extends a relatively short distance, roughly the width of an adjacent cell.^2,10,15^ Thus, at confluence, the cells sense the mechanical environment of surrounded cells in conjunction with that of the underlying substrate. In turn, this could counterbalance the influence of the underlying stiffness. Additionally, cell–cell signaling can regulate cell function, attenuating the effect of the mechanical environment. For this reason, the subconfluent cell density was used to show the direct effect of ECM environmental characteristics on cell behavior. The higher levels of cell-peripheral PECAM-1 on peptides compared to Col I coatings suggest that the high density of integrin-mediated signaling provided by small, high affinity GFOGER and GLOGEN peptides can drive the formation of PECAM-1-containing cell–cell junctions.

By contrast to HUVECs, peripheral PECAM-1 localization in HDMECs, at either confluence or subconfluence, was largely insensitive to mechanical cues. Instead, these cells were very sensitive to the coating biomolecule, with significantly higher levels of peripheral PECAM-1 on peptides compared to Col I surfaces. Moreover, HDMECs were highly sensitive to the amino acid sequence of the cell-binding motif contained within these peptides, clearly exhibiting increased PECAM-1-containing cell–cell junctions on GFOGER compared to GLOGEN. This suggests that ligand affinity toward their respective integrin receptors was crucial for stimulating the formation of PECAM-1-containing cell–cell junctions. Of the collagen-binding integrins, HDMECs predominantly express integrin α2β1 (unpublished data) for which the triple-helical GFOGER motif represents the highest affinity ligand. Therefore, the elevated PECAM-1-containing connections on GFOGER coatings could be a function of integrin affinity and subsequent signaling. The relative insensitivity of HDMECs to the substrate stiffness may be due to the intrinsic mechanosensing properties of HDMEC’s or alternatively may be a consequence of cell signaling from collagen-binding receptors compensating for mechanotransductive signaling.

Cells can perceive the mechanics of the ECM through numerous mechanisms. A nonexhaustive list includes via p130^CAS^,^7^ vinculin recruitment to talin-containing FA,^6^ focal adhesion kinase (FAK) signaling,^5^ linker of nucleoskeleton and cytoskeleton (LINC) complexes,^77^ G-protein coupled receptors (GPCRs),^78^ ion channels,^79^ and force-induced exposure of cryptic sites on the ECM molecules.^8^ Further investigation to determine the relative contribution of these mechanisms to the stiffness-induced cell morphological effects observed here would add significant insight.

## 5 Conclusions

In this work, we use a model system where surface rigidity was varied across a physiologically relevant range while independently controlling cell-surface integrin engagement. We showed that integrin adhesion could be controlled completely separately from substrate stiffness. Using this experimental tool, we analyzed the response of different cell types to changes in the mechanical environment and the presence of collagen-derived cell-ligating motifs. We found that the use of triple-helical peptides (GFOGER and GLOGEN) enhanced the effect of rigidity on the cellular response compared to using fibrillar collagen coatings. Integrin-dependent HDF adhesion was largely insensitive to changes in matrix stiffness, while cytoskeletal organization and development of the mechanosensitive complexes such as SF and FA were promoted on stiffer substrates. The initial adhesion of HUVECs and HDMECs was affected by the material modulus with increased adhesion to peptides on lower stiffness surfaces. By comparison, the localization of PECAM-1 to cell–cell junctions was dependent on both substrate mechanics and the type of biomolecule bound to the surface. The fact that different aspects of cellular behavior can be influenced by substrate rigidity highlights the importance of including mechanical cues when considering the cell response to macromolecular materials. This, in turn, can be used to direct cell behavior by tuning the mechanical properties of cellular supports aimed at selected applications.

## Supplementary Material



## Data Availability

The underlying data for this article may be found at 10.17863/CAM.XXXXX.

## Notes

Flowchart of the image processing steps used to quantify the PECAM at the periphery of the endothelial cells (PDF)

## †

Laboratory of Tissue Biology and Therapeutic Engineering, CNRS UMR 5305, University Claude Bernard-Lyon 1 and University of Lyon, 7 Passage du Vercors, Cedex 07, Lyon 69367, France

## Φ

Triple Helical Peptides Ltd., Cambridge, CB22 5DU, United Kingdom.
