# Supplementary material for: Impact of Stiffness and Cell-Binding Motif Availability on the Cell-Specific Response to Collagen-Based Macromolecular Materials
Source: Biomacromolecules. 2026 May 25;27(6):3501–16. doi: 10.1021/acs.biomac.5c01434 (PMC13250905; doi:10.1021/acs.biomac.5c01434)

Impact of stiffness and cell-binding motif availability on the cell-specific response to collagen-based macromolecular materials.

Natalia Davidenko[1#], Daniel V.Bax[1#*], Emma Hunter [2], Samir W.Hamaia[2], Jean-Daniel Malcor[2†], Richard W.Farndale[2Φ], Sanjay Sinha[3], Serena M. Best[1], Ruth E.Cameron[1]

[1] Department of Materials Science and Metallurgy, University of Cambridge, Cambridge, CB3 0FS, United Kingdom

[2] Department of Biochemistry, University of Cambridge, Cambridge, CB2 1QW, United Kingdom

[3] Department of Medicine, Cambridge Stem Cell Institute, University of Cambridge, Cambridge, CB2 0AW, United Kingdom

[†]Current address: Laboratory of Tissue Biology and Therapeutic Engineering, CNRS UMR 5305, University Claude Bernard-Lyon 1 and University of Lyon, 7 Passage du Vercors, Cedex 07, Lyon 69367, France

[Φ] Triple Helical Peptides Ltd, Cambridge, CB22 5DU, United Kingdom,

# These authors contributed equally

* Corresponding author: dvb24@cam.ac.uk

Supplementary figure 1 - PECAM1 quantification flow chart

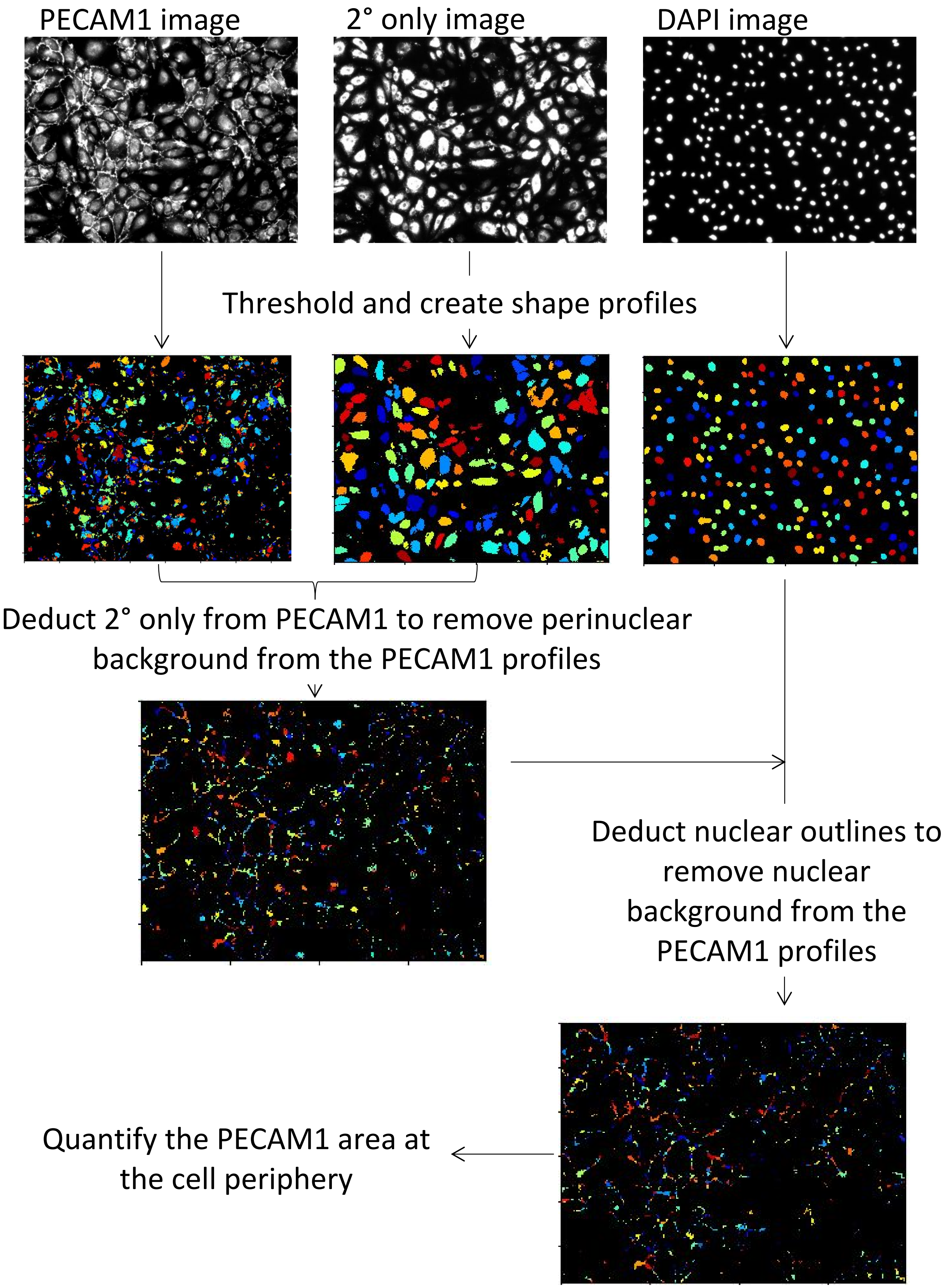

Supplement: Supplementary file 1 [file bm5c01434_si_001.pdf]
